# A novel semi-supervised framework for UAV based crop/weed classification

**DOI:** 10.1371/journal.pone.0251008

**Published:** 2021-05-10

**Authors:** Shahbaz Khan, Muhammad Tufail, Muhammad Tahir Khan, Zubair Ahmad Khan, Javaid Iqbal, Mansoor Alam

**Affiliations:** 1 Department of Mechatronics Engineering, University of Engineering & Technology, Peshawar, Pakistan; 2 Advanced Robotics and Automation Laboratory, National Center of Robotics and Automation (NCRA), Rawalpindi, Pakistan; 3 College of Electrical & Mechanical Engineering (CEME) National University of Sciences and Technology (NUST), Islamabad, Pakistan; Taipei Medical University, TAIWAN

## Abstract

Excessive use of agrochemicals for weed controlling infestation has serious agronomic and environmental repercussions associated. An appropriate amount of pesticide/ chemicals is essential for achieving the desired smart farming and precision agriculture (PA). In this regard, targeted weed control will be a critical component significantly helping in achieving the goal. A prerequisite for such control is a robust classification system that could accurately identify weed crops in a field. In this regard, Unmanned Aerial Vehicles (UAVs) can acquire high-resolution images providing detailed information for the distribution of weeds and offers a cost-efficient solution. Most of the established classification systems deploying UAV imagery are supervised, relying on image labels. However, this is a time-consuming and tedious task. In this study, the development of an optimized semi-supervised learning approach is proposed, offering a semi-supervised generative adversarial network for crops and weeds classification at early growth stage. The proposed algorithm consists of a generator that provides extra training data for the discriminator, which distinguishes weeds and crops using a small number of image labels. The proposed system was evaluated extensively on the Red Green Blue (RGB) images obtained by a quadcopter in two different croplands (pea and strawberry). The method achieved an average accuracy of 90% when 80% of training data was unlabeled. The proposed system was compared with several standards supervised learning classifiers and the results demonstrated that this technique could be applied for challenging tasks of crops and weeds classification, mainly when the labeled samples are small at less training time.

## Introduction

Agriculture plays a vital role in the global economy and has led to extensive studies to increase crop yield [1, 2]. Weeds pose a severe threat in achieving this goal, as it accounts for about a 35% reduction of global crop yields [3]. Agrochemicals (Herbicides and Pesticides) are used for controlling weed infestation and allow the efficacy of almost 75% [3, 4]. These chemicals are typically broadcasted over the entire fields, including weed-free regions with economic and environmental risks associated with them [3, 5]. Site-specific weed management (SSWM) recommends chemical saving by utilizing an adequate amount of chemicals based on weed density [4]. In this regard, an accurate classification system of crops and weeds could significantly promote chemical saving while enhancing its effectiveness by providing decision-making information for variable-rate spraying machines. This classification system is obtained either by ground sampling or by remote detection [3]. The reliability of remote sensing fields is significantly higher than ground visits [4, 6].

Rapid advancement in Unmanned Aerial Vehicle (UAV) technology for several civilian applications makes it an ideal choice for remote sensing in precision agriculture applications [7, 8]. Compared to other platforms for remote sensing (aircraft, satellite, etc.) UAV offers a more cost-effective and efficient solution due to low altitude flights and the ability to capture high-resolution imagery for monitoring small weed patches thoroughly [9–12]. Extensive work has been carried in the literature on UAV for different precision agriculture applications (weed mapping, crop management, spraying, etc.) [3, 13–18] Weed detection and recognition in early growth stages for dicotyledonous (pea and strawberry etc.) crops are much more challenging as compared to late-stage [19] due to the following three reasons: 1) The dicotyledonous crops and grass weeds usually have similar reflectance characteristics, which is very difficult to be differentiated through RGB imagery, 2) weeds are generally distributed in small patches, 3) interference of soil background reflectance with the detection.

Conventionally human craft features such as color, shape, and texture are used for early-stage detection [20], but they are prone to errors. They have led to the introduction of deep learning in weed classification and recognition [21–23]. However, a large amount of training data is essential for this technique. Collecting specific label images is a challenging and time-consuming task and needs to be addressed.

Furthermore, the training time and processing time required for these techniques is relatively high and is not ideal for UAV platforms due to their computational constraint. One potential approach for improving the recognition accuracy with limited data set is deploying semi-supervised learning, and the study aims to extend this by using a generative adversarial network (GAN) [24–26] to accurately classify the RGB imagery obtained of early growth crops through UAV into weeds and crops with few label images. The main contribution of this research are, i) Modified semi-supervised GAN (SGAN), as the images in the study had a 448 * 448-pixel resolution compared to conventional 28 * 28 resolution, ii) to apply SGAN to real RGB imagery iii) to achieve less training time for lower labeled rate iv)to analyze the potential of using this technique in the field by applying it at least two different fields and v) motivate learning from unlabeled training data by comparing the accuracy with conventional supervised learning classifiers.

The remaining of this paper is organized as follows: Related work is explained briefly in Section 2; Section 3 discusses Materials and Method followed by experimentation in Section 4 Materials and; Section 5 discusses the results followed by discussion, and finally, Section 6 conclude this article.

## Related work

Annotating large datasets for classification systems is a concern that becomes even more challenging when the number of unsupervised samples for training the classifier is more than the supervised samples [27]. In this regard, semi-supervised learning methods learn from the partially labeled data set and improve classification performance. Normally semi-supervised learning techniques either employ one by one supervised classification for propagating labels to unlabeled training samples [27–29] or explore the spatial distribution of the samples in feature space for prorogation [27]. The path-based similarity has also been applied [30] to capture the manifold structure data and obtain similarity for maximizing the separability between various classes. Currently, deep learning is mostly applied for classification tasks by employing complex neural architectures [31]; however, employing unsupervised data could be beneficial for improving classification performance [32, 33]. Recent advancements in this field have proposed using unsupervised samples to enhance learning in a deep neural network. Unlabeled samples were employed for pre-training the network, which was later adjusted by using labeled data [33, 34]. In a semi-supervised learning method proposed for deep neural networks [35] that can learn both labeled and unlabeled samples simultaneously, SoftMax outputs were utilized for pre-labeling the unlabeled samples. In another instance, a weak semi-supervised deep learning technique was proposed that incorporated Convolutional Neural Networks (CNNs) for annotating multiple label images [36]. In their study, weak labeled or even label-less images were applied to train the network.to manage the weighted pairwise ranking loss was utilized to manage the weakly labeled images.

In contrast, for unlabeled images, triplet similarity loss was applied. In another study, a system was developed for weed mapping in sunflower crops for site-specific weed control treatments [3]. Different degrees of labeled data were utilized to explore different machine learning algorithms, and the results showed that a semi-supervised system was able to adapt well, even when a few labeled training samples were considered. In the subject of active learning, CNN was used to develop a semi-supervised algorithm [37] to find representative unlabeled samples and the regularization term, which was new in the loss function. A pseudo labeling approach was developed and employed [38] for semi-supervised learning for classifying hyperspectral images—the developed method used pseudo-labeled samples for training a deep recurrent CNN. The proposed technique outperformed the recent supervised and semi-supervised learning methods for classifying hyperspectral images. A semi-supervised method was employed in a study to classify an unlabeled training set, which was used for training a CNN [39]. The technique was validated with soybean leaf and pest identification from images obtained through UAV to classify unlabeled training set, which was applied for training a CNN. The technique was validated with soybean leaf and pest identification from images obtained through UAV. Albeit, extensive work has been carried out in semi-supervised learning as explained in this section but still, there is room for improvement, however, particularly in cases where the classification poses a challenge like classifying crops and weeds in the early growth stage when the spectral and appearance characteristics are similar. Semi-supervised generative adversarial network (SGAN), in which generator develops large realistic images, which in turn forces the discriminator to classify accurately by learning better features, achieved good accuracy [25], [26] or classification tasks. So far, SGAN application has not been explored for classifying Red Green Blue (RGB) imagery of early growth stage crops and our study aims to address this research gap, by using SGAN for classifying crops and weeds in early growth croplands, using UAV RGB imagery.

## Materials & methods

### Semi-Supervised Generative Adversarial Network (SGAN)

**Fig 1:**
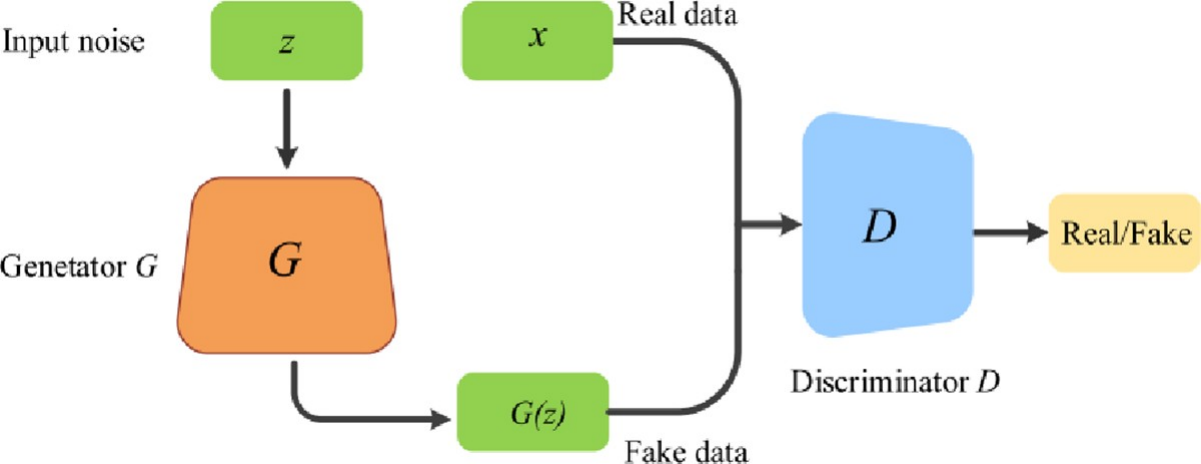
Architecture of GAN [41].

Generative Adversarial Network (GAN) frameworks were first introduced for training deep generative models using game theory [40]. It contains two models: generator (G) and discriminator (D), both are trained in an adversarial manner. Fake inputs are generated by the G, while discriminator (D) classifies real and fake inputs [25, 41] as illustrated in Fig 1.

Both generator and discriminator are trained to optimize the results, i.e., D accurately classifies generator distribution from real data. At the same time, G generates data possessing similar distribution like actual data to fool the discriminator into accepting generator output as real data [42]. GANs have been employed in different applications such as image generation, classification, etc. [26]. Recently GANs have been extended into semi-supervised learning for image classification [25, 26, 39]. Usually, multiclass classifier labels an input into one of the K discrete classes. For semi-supervised learning using GAN, a new label K+1 could be added for the samples generated by G corresponding to fake class [26]. Unlabeled data could be used for learning [42] by defining loss function for discriminator. Supervised and unsupervised loss function combines to form the discriminator loss function as follows [43].

$$L=L_{supervised}+L_{unsupervised}$$ (1)

$$L_{supervised}=-E_{x,y∼p_{data}(x,y)}{{log}_{p}}_{model}(y|x,y<K+1)$$ (2)

The supervised loss function (L_supervised_) is defined as the negative log probability of y when the correct class is allocated by x. L_supervised_ focuses on correctly classifying material to a given labeled sensor measurement x

$$L_{unsupervised}=-E_{x,y∼p_{data}(x_{u})}\log\left(1-p_{model}(y=K+1|x_{u})\right)-E_{x∼G(z)}^{∼}\log p_{model}(y=K+1|x^{∼})$$ (3)

Unlabeled image loss functions constitute the unsupervised loss function (L_unsupervised_). *p_model_*(*y* = *K*+1|*x*) represents the probability that x is fake, corresponding to 1-D(x) of GAN architecture [26]. Unlabeled data sample is denoted by x_u_. The unlabeled sensor measurement is classified to one of the K classes by the first term of L_unsupervised_. The second term in the L_unsupervised_ classifies the images generated by the G as K+1 (fake) [26, 42].

By minimizing L_supervised_ and L_unsupervised_ the classifier could be trained with gradient descent. The D is updated stochastically at each training iteration by gradient descent of the Eq 1.

$$\nabla_{\theta_{d}}1/m\sum_{i=1}^{m}-\log\sigma {(x}^{\left(i\right)})y^{\left(i\right)}-\log D(x_{u}^{i})-\log(1-D(G(z^{\left(i\right)})))$$ (4)

For all m samples in a minibatch σ(x)_j_ = *p_model_*(*y* = *j*|*x*) (SoftMax function) applied at the output of D.

**Fig 2:**
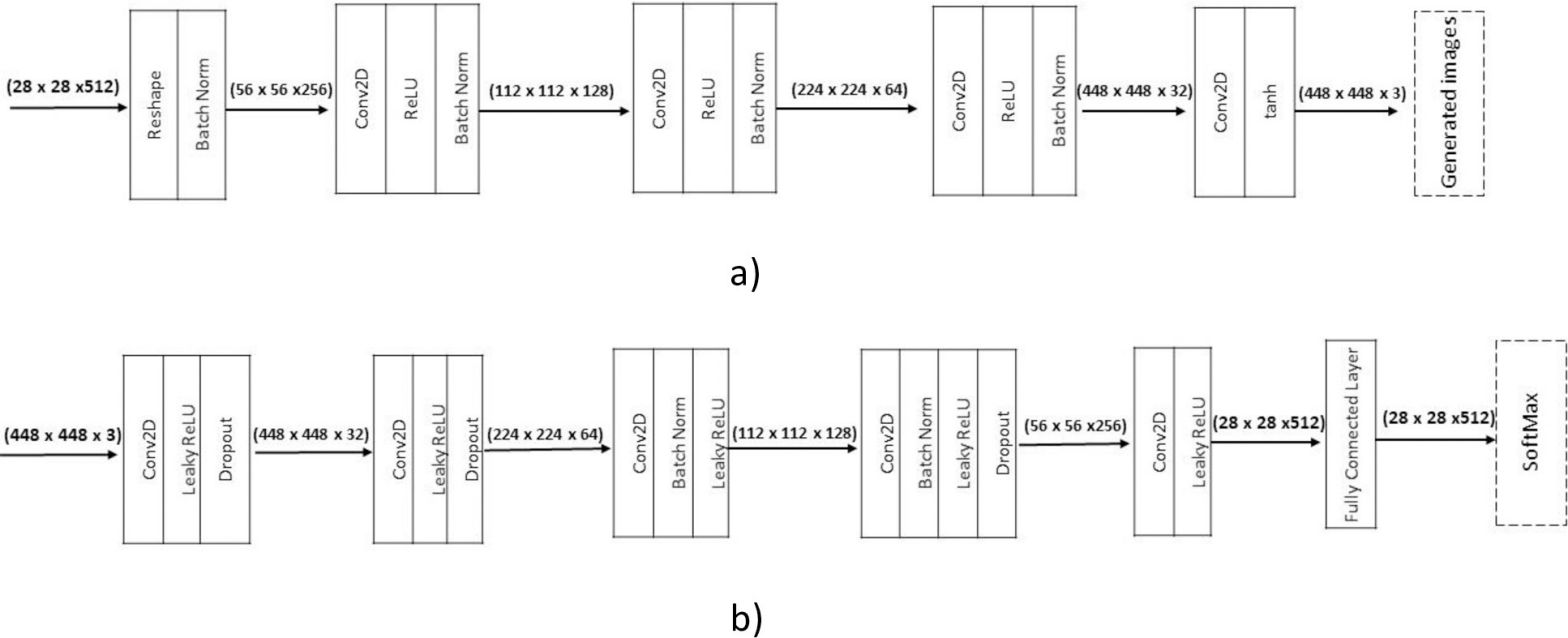
Structure of modified SGAN. a) Generator. b) Discriminator.

The conventional SGAN was designed to deal with a 28* 28-pixel image. During preliminary testing the images were rescaled to the traditional pixel size. However, the results were not satisfactory as images suffered an information loss, which is of utmost importance in achieving the desired accuracy. Therefore, the SGAN is modified to use 448*448 images which are illustrated in Fig 2.

**Fig 3:**
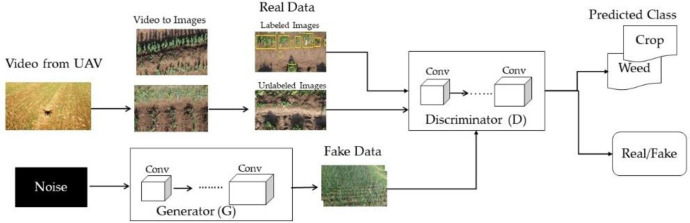
Overview of proposed methodology.

The video was recorded at the preprocessing stage and was converted into images. The images were divided into two batches of labeled and unlabeled images. The proposed model generator took noise distribution as an input, and after four convolution layers generated a fake image like real distribution data. The discriminator input layer size was changed to 448*448*3, and the reshape layer of the generator was adjusted to 28*28*512, followed by four convolutional layers, which up sampled the random noise in each layer for producing 448*448 images. The size of convolutional kernel for G was 5*5. Rectified Linear Unit (ReLU) activation function was applied to all the layers of a generator except the output, which used the Tanh. The standard Adam optimizer was employed for D and G, and the learning rate was set to 0.00001. The generated images, along with real data images (labeled and unlabeled), were processed by the D, a fully convolutional multiclass classifier was utilized instead of the conventional D. The size of convolutional kernel was 3*3. The classifier could predict the input data to a label y from two K classes (crops and weeds) or to a fake sample (k+1 class). The main idea was obtained through [44] to add large fake images forcing the real samples close in feature space resulting in improved classification. SoftMax was utilized in the last layer for the discriminator for enabling classification. The overview of the proposed method is illustrated in Fig 3. The training of the proposed SGAN is shown in Algorithm 1.

**Algorithm 1 Proposed SGAN Training**

**Input:** labeled samples {(x^1^,y^1^),…(x^m^,y^m^)}, Unlabeled samples {u^1^,…u^m^}, Number of iterations, Generated data, Fake data, Real data

**Initialize:** Labeled samples = X_Label_, Unlabeled samples = X_unlabel_, I = Number of iterations, Generated data = X_G_, Fake data = X_F_, Real data = X_R_

**for *i* = 1 to, *I do***

            Normalize input data [–1 1]

    **Training Discriminator (D):**

    Freeze generator weights

    Train ***D*** with X_L_, Calculate L_supervised_ *(Eq 1)*

    Train ***D*** with X_L_, X_UL_, X_G,_ Calculate L_unsupervised_ *(Eq 2)*

    L_D_ = L_supervised_ + L_unsupervised_

    Update D weights by their gradient

    $\nabla_{\theta_{d}}1/m\sum_{i=1}^{m}-\log\sigma {(x}^{\left(i\right)})y^{\left(i\right)}-\log D(x_{u}^{i})-\log(1-D(G(z^{\left(i\right)})))$

    **Training Generator (G):**

      Freeze D weights

      Feed D with half of X_R_ and X_F_

      Extract features and Calculate $L_{G}={\left|E_{x{∼}_{preal}}f(x)-E_{x{∼}_{fake}}f(x)\right|}_{2}^{2}$

        Update G weights by their gradient

        $\nabla_{\theta_{g}}[1/m\sum_{i=1}^{m}|f(u^{i})-f(G(z^{i})){|}_{2}^{2}$

            **End for**

**____________________________________________________________________________________________**

## Experimentation

### Data acquisition

**Fig 4:**
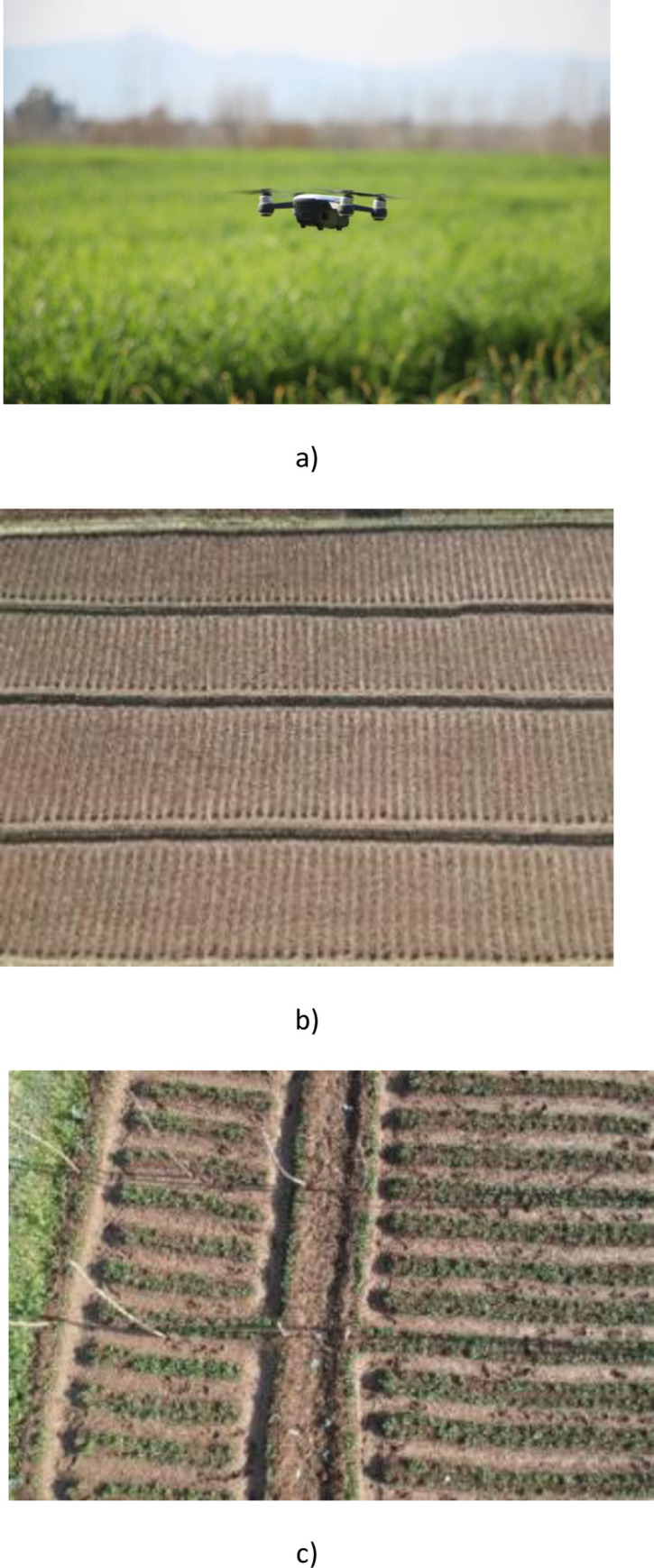
Overview of UAV and experimental site. a) DJI Spark (Multi-Rotor). b) Pea Field. c) Strawberry Field.

Da-Jiang Innovations (DJI) Spark having an onboard camera, was employed for collecting images with 1/2.3" CMOS sensor, and FOV 81.9° 25 mm f/2.6 lens was utilized for collecting imagery as depicted in Fig 3A. The experimental site was located at Turangzai city, Peshawar, Khyber Pakhtunkhwa (KP), Pakistan, Coordinates 34° 12’ 57" North, 71° 44’ 50" East). Two different croplands, pea, and strawberry were selected with an area of 0.4 ha and 0.2 ha shown in Fig 4B and 4C. The seeds of strawberry and pea were sown on December 15, 2019, and January 20, 2020, respectively. The area was naturally infested with inter and intra row annual goosegrass (Eleusine indica) weeds after 15 and 25 days respectively.

**Fig 5:**
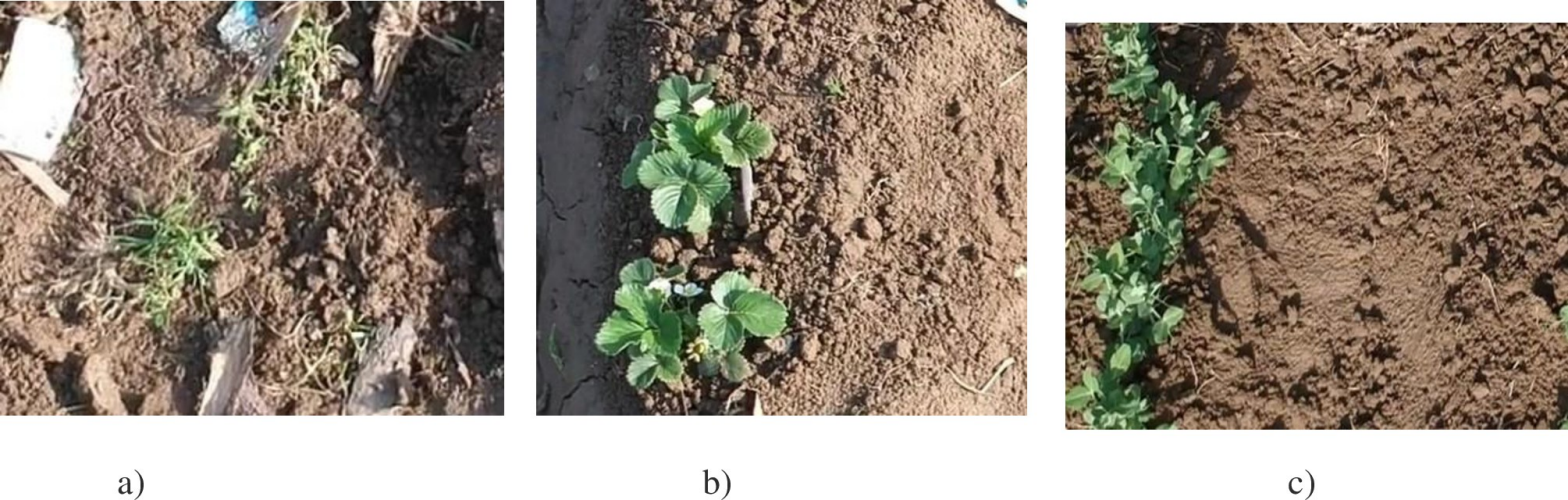
Sample images for the crops (pea and strawberry) and weeds dataset. a) Weeds. b) Crops. c) Crops.

Flights were conducted for one week from February 23 to March 1 2020, just when both the crops and infested weeds were in the early growth stage, and agrochemical treatment was recommended. To ensure uniform lighting, all images were collected in the morning hours, i.e., during the daytime. The average temperature was ranged between 23–25 ^○^C while ambient humidity was 65–68%. A rectangular region of 50*40 m was selected for conducting the flight operations. The altitude for the flight was kept 2m above the ground, and spatial resolution was 0.3cm. UAV collected videos, which were converted into images using a Joint Photographic Group (JPG) converter. Images obtained through the first half of the video were used for training, while the latter half of the video was used for testing. Two classifier data sets i.e., crop (pea and strawberry) and weeds, were collected. The data set comprised 2600 images and 2800 images of crops and weeds, each for pea and strawberry, respectively. Fig 5 shows a sample of labeled images for the developed dataset of crops and weeds. The data set was split into 70% for training, 15% each for validation and testing. Images of the training set were labeled manually on an experimental platform. Labeler consensus was developed, and auditing was performed through the field experts for counteracting any error in the labeling. The accuracy of labeling work was 99%, and the confidence score was 0.98. Tensor flow and Keras open-source deep learning framework were used for experimentation, while the platform used was Intel i7-6600U processor, 8GB DDR4 memory, AMD Radeon R7 M360 2GB graphics card. The hyper-parameter settings were: batch size = 30; learning rate = 0.0001. The moment of 0.8 and weight decay of 0.0005 was utilized.

## Results and discussion

**Fig 6:**
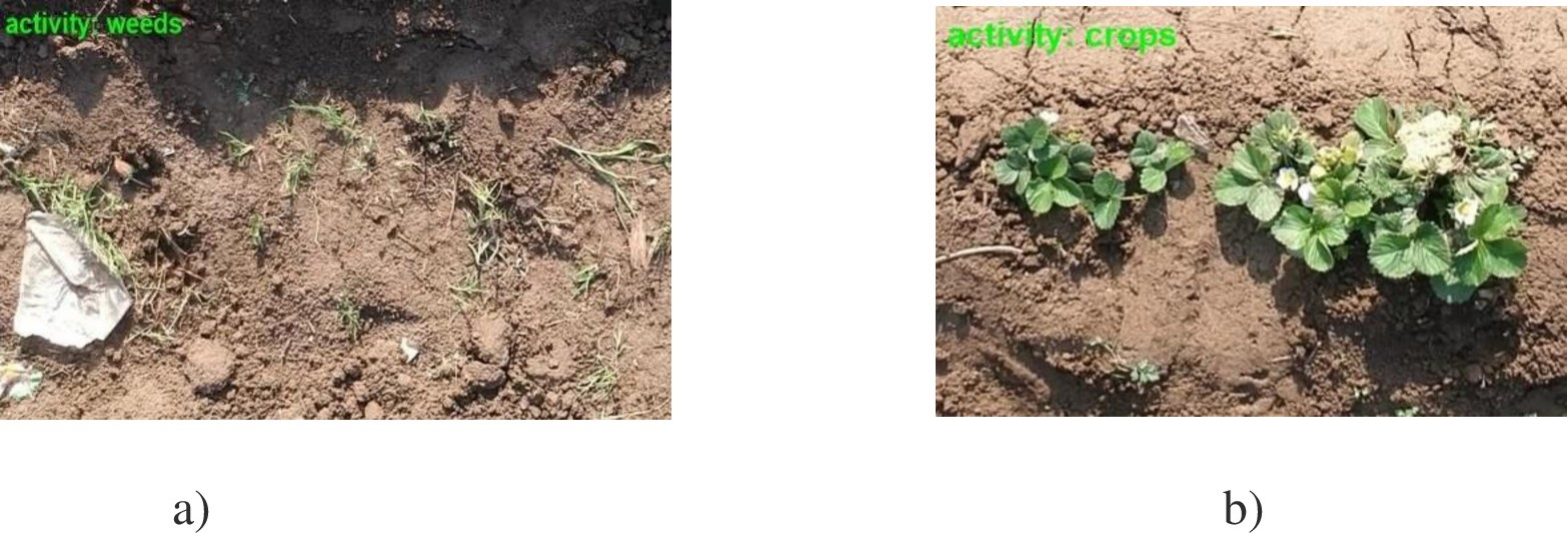
Crops and weeds classification for the developed system. a) Weeds. b) Crops.

After rigorous experimentation, the results achieved are presented in this section. A specific number of labeled and unlabeled samples for both the croplands (illustrated in Table 1) was used as an input to the discriminator for semi-supervised training. L was used for denoting the labeled images, while U was used for the unlabeled data set. The classification of crops and weeds with their for the developed method is illustrated in Fig 6.

**Table 1:** Number of the labeled and unlabeled Images under different labeled rates for two different croplands.

Crop Land (Pea)			
Labeled Rate	Labeled Images (L)	Unlabeled Images (U)	Total
20%	260	1040	1300
40%	520	780	1300
60%	780	520	1300
80%	1040	260	1300
Crop Land (Strawberry)			
Labeled Rate	Labeled Images (L)	Unlabeled Images (U)	Total
20%	280	1120	1400
40%	560	840	1400
60%	840	560	1400
80%	1120	280	1400

The proposed method’s training time for each labeled rate was calculated by finding an average time from the 800^th^ epoch to the 1000^th^ epoch and is shown in Table 2. The labeled rate is directly proportional to the training time, which means that as the labeled rate increases, the training time also increases. The conclusion is per the desired objective of the developed framework.

**Table 2:** Average training time at different labeled rates.

Labeled Rate	Average Training Time (Sec/Epoch)	Total Epochs
20%	38.96	1000
40%	49.93	1000
60%	50.88	1000
80%	51.32	1000

**Fig 7:**
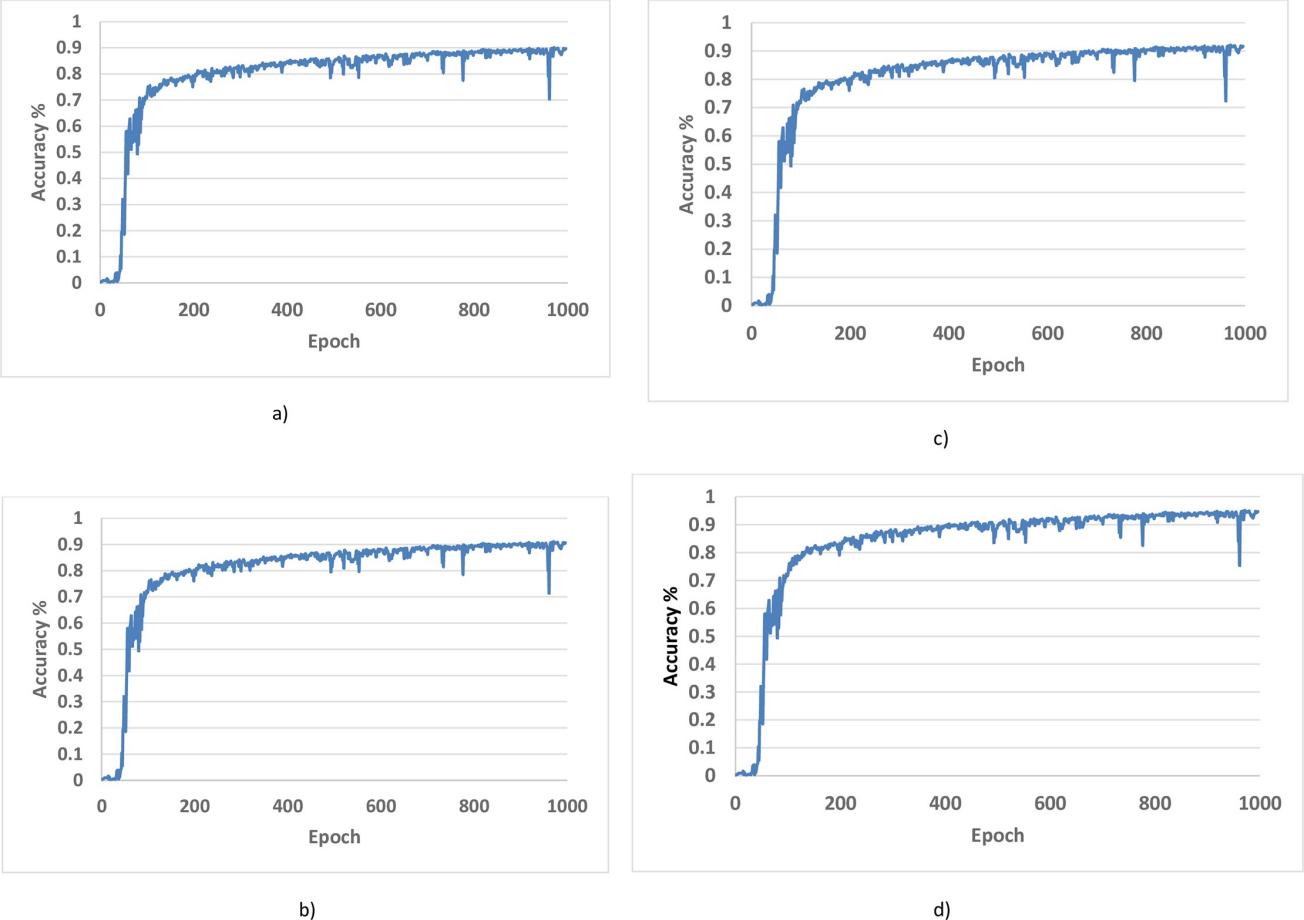
Cross validation accuracy (SSCA) using different labeled rates. (a) Labeled Rate 20% b) Labeled rate 40% c) Labeled rate 60% d) Labeled rate 80%.

Cross-validation accuracy for 1000 epochs for all the labeled rates is shown in Fig 7A–7D. It is evident that the accuracy increases with an increase in the number of epochs. The training has not reached a plateau even at the 1000 epoch, which shows that further training can enhance accuracy.

Labeled and unlabeled samples were used simultaneously for semi-supervised training and obtained semi-supervised classification accuracy (SSCA), which is expressed in terms of the following equation from a confusion matrix

$$SSCA(labeled+Unlabeled)=\frac{True\;Positive\;(TP)+True\;Negative\;(TN)}{Positive+Negative}$$ 5)

Table 3 shows quantitative results for SSCA on the data mentioned above. Accuracy based on confusion matrix was used as an evaluating metric.

**Table 3:** Classification accuracy of our proposed semi-supervised learning method under different labeled rates.

Crop Land (Pea)				
	Labeled Rate %			
Target	20% Labeled	40% Labeled	60% Labeled	80% Labeled
	L+U	L+U	L+U	L+U
	SSCA	SSCA	SSCA	SSCA
Crops and Weed	89.15%	90.08%	91.36%	93.75%
Crop Land (Strawberry)				
Target	Labeled Rate %			
	20% Labeled	40% Labeled	60% Labeled	80% Labeled
	L+U	L+U	L+U	L+U
	SSCA	SSCA	SSCA	SSCA
Crops and Weed	90.06%	90.66%	91.98%	94.6%

The developed semi-supervised method achieved an overall accuracy of almost 90% for two croplands when the labeled samples were only 20%. It performed better classification performance when the labeled samples were increased and achieved an overall accuracy of 94.17% when 80% of the samples were labeled; it is because that the proposed semi-supervised GAN might result in incorrectly labeled samples, which made it impossible for newly labeled images to perform similarly to the original labeled samples.

### A comparison for evaluation

It was essential to validate the developed technique, for which a comparison was made with well-established classification techniques such as Support Vector Machine (SVM) [45], K-Nearest Neighbor (KNN) [46], CNN [47]. SVM, working on the principle of statistical learning theory, is a machine learning method that uses nonlinear kernel functions for mapping original input data into high dimensional features seeking separate hyperplane, and the data is optimally separated into two categories by using the constructed N-dimensional hyperplane [45]. KNN is another standard machine learning algorithm that uses neighbor votes for classifying objects, resulting in assigning objects to the most common class. K represents a small positive value. The nearest neighbor class is assigned when the value of the object is 1. Euclidean distance is a commonly used metric for calculating the closest k elements distance [46]. CNN is a highly accurate technique normally used to classify images [48]. CNN has multiple architectures (LeNet5, Alex Net, VGG 16, etc.), providing different results. The architecture used in the study for comparison are LeNet5, VGG-11, VGG-16, ResNet18, and ResNet50. VGG-11 and VGG -16 comprises eight (8) and thirteen (13) convolutional layers, followed by three (3) fully connected layers and a single SoftMax layer [47]. LeNet5 is a classical CNN model consisting of seven convolutional layers [47]. Whereas, ResNet18 and ResNet50 comprises of 18 and 50 convolutional layers followed by batch normalization and a ReLU activation function [49].

The labeled samples were only used for obtaining supervised classification accuracy (SCA) with the techniques mentioned above. Quantitative results for SCA are obtained and illustrated in Table 4.

$$SCA(labeled)=\frac{True\;Positive\;(TP)+True\;Negative\;(TN)}{Positive+Negative}$$ 6)

**Table 4:** Supervised classification accuracy (SCA) of KNN, SVM, LeNet5, VGG-11VGG-16, ResNet18, and ResNet50 on different labeled rates.

Cropland (Pea)				
Method	Labeled Rate			
	20%	40%	60%	80%
KNN	72.14%	75.22%	79.23%	83.21%
SVM	54.25%	57.20%	62.55%	65.15%
LeNet5	85.12%	88.61%	91.46%	94.11%
VGG-11	85.73%	88.92%	91.89%	94.93%
VGG-16	86.24%	89.13%	92.23%	95.01%
ResNet18	87.11%	89.42%	92.47%	95.63%
ResNet50	87.67%	89.69%	92.83%	95.98%
Cropland (Strawberry)				
Method	Labeled Rate			
	20%	40%	60%	80%
KNN	73.05%	75.74%	79.89%	84.92%
SVM	55.34%	58.42%	64.04%	66.43%
LeNet5	86.04%	88.31%	92.01%	95.03%
VGG-11	86.71%	88.83%	92.63%	95.79%
VGG-16	87.13%	89.37%	93.08%	96.21%
ResNet 18	87.51%	89.77%	93.47%	96.53%
ResNet 50	88.03%	89.91%	93.89%	96.92%

**Fig 8:**
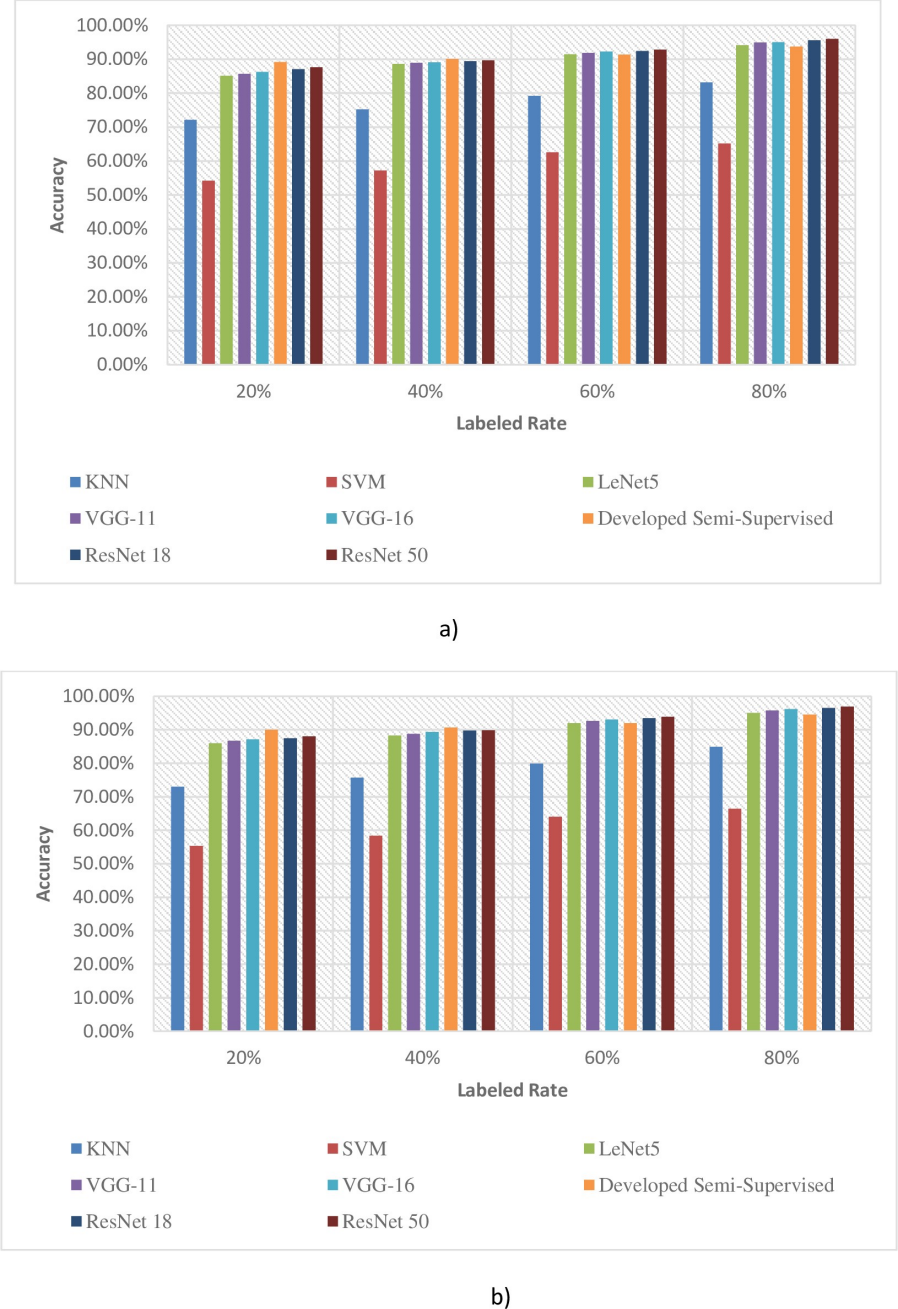
Comparison between SCA and SSCA at different labeled rates. a) Pea. b) Strawberry.

The comparison between the SCA and SSCA on different labeled rates is shown in Fig 8.

For the Pea cropland, the developed semi-supervised system performed consistently well, as compared to the KNN system with at least 10% superiority across all ranges of labeled images; while this superiority was even more when compared to the SVM system, with a minimum improvement in accuracy of around 28% even at (80% labeled images), while yielding more than 34% superiority at 20% labeled images.

For the remaining systems, i.e., LeNet5, VGG-11, VGG-16, ResNet18, and ResNet50, developed proposed system performed in a consistently superior manner at both 20% and 40% labeled images; however, the remaining methods proved to be marginally superior (around 2% max.) at 80% labeled images.

**Fig 9:**
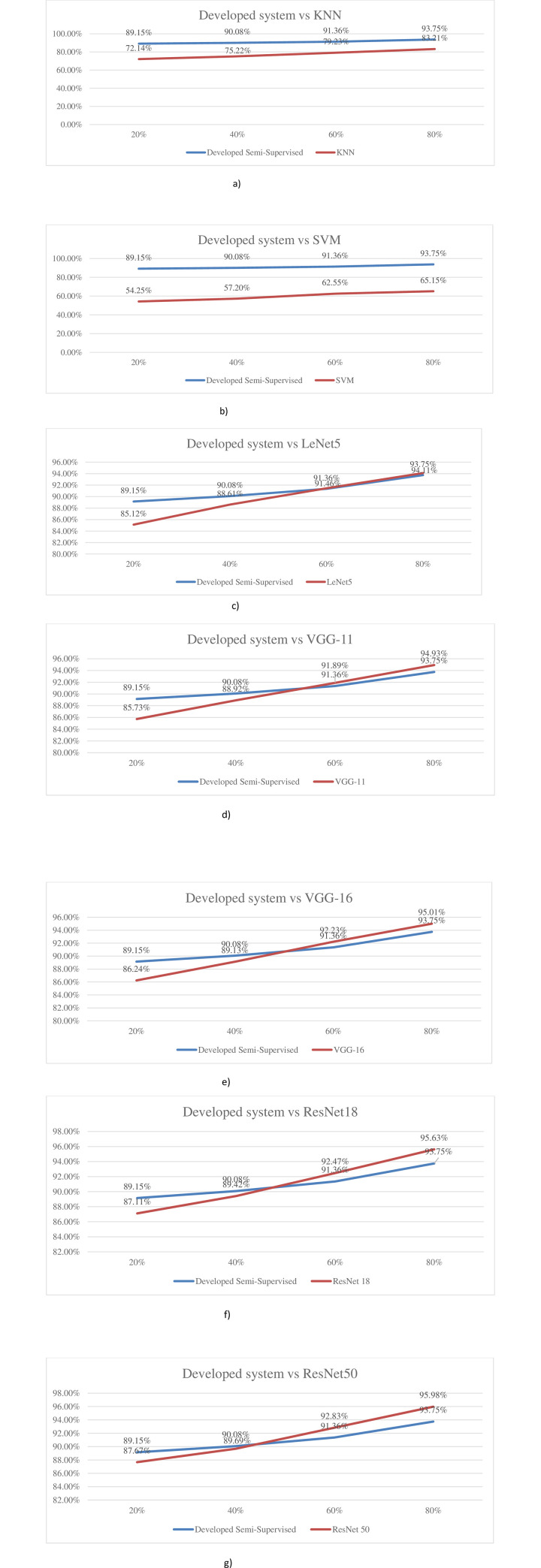
Comparison of developed system with individual systems. a) Developed system vs KNN. b) Developed system vs SVM. c) Developed system vs LeNet5. d) Developed system vs VGG-11. e) Developed system vs VGG-16. f) Developed system vs ResNet18. g) Developed system vs ResNet50.

The inferences are evident from the comparisons appended below (Fig 9A–9G) regarding the developed system against each system across the four different percentages of labeled images.

Similar to the results for Peas, considering the Strawberry cropland, developed semi-supervised system also performed consistently well in almost an identical manner (removing any bias/ differences on account for different croplands), as compared to the KNN system with more than 9.7% superiority across all ranges of labeled image percentages. As expected, the superiority was even more when compared to the SVM system with a minimum superiority of again 28% superiority in identification accuracy (even at 80% labeled images), while yielding almost identical 34.7% superiority at 20% labeled images.

For the remaining systems, i.e., LeNet5, VGG-11, VGG-16, ResNet18, and ResNet50, the developed system performed in a consistently superior manner at both 20% and 40% labeled images; however, the remaining methods also proved to be marginally superior (around 2.3% max.) at 80% labeled images.

**Fig 10:**
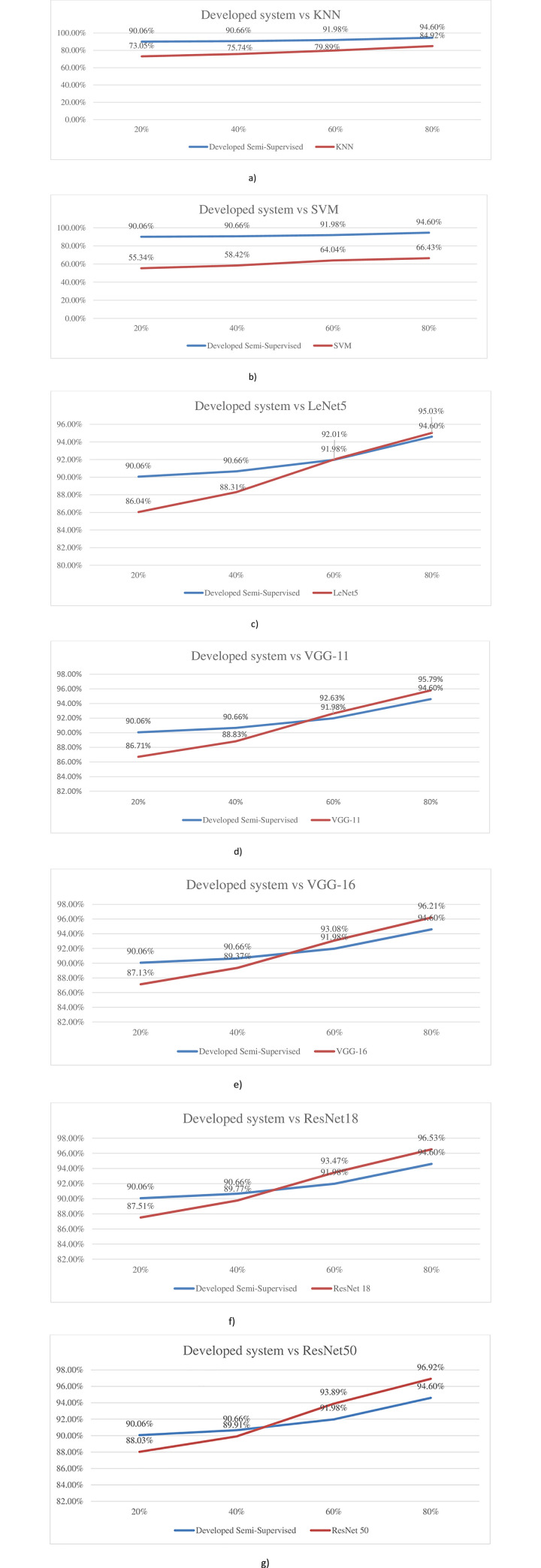
Comparison of developed system with individual systems for strawberry. a) Developed system vs KNN. b) Developed system vs SVM. c) Developed system vs LeNet5. d) Developed system vs VGG-11. e) Developed system vs VGG-16. f) Developed system vs ResNet18. g) Developed system vs ResNet50.

The inferences are again evident from the comparisons appended below (Fig 10A–10G) regarding the developed system against each system across the four different percentages of labeled images.

The study demonstrated that in cases where the available data is limited, the developed technique could achieve classification with comparatively good accuracy across several classification applications. One particular application being studied in this study is crop weed classification, which is an essential area in precision agriculture applications, as this would ensure a platform for precise spraying of pesticides. This application may prove significant in overcoming the socioeconomic impact (millions of dollars lost) on the global economy, especially in developing countries. Furthermore, the broadcast (conventional) spraying has several limitations on the environment and the crops. In this regard, a robust semi-supervised system was developed for classifying crops and weeds accurately, even with limited labeled data. The developed method has the potential to be used as a transfer learning technique and can be deployed to train crops and weeds classifier for other horticulture crops (fruits and vegetables). Additionally, the experimental results indicate that the developed framework can provide decision-making support to specialists, farmers, precision agriculture sprayers (tractor, UAV, etc.) for the site-specific weed management (SSWM) operations. Once extended to a precision agriculture sprayer operating situation, the developed system would lead to substantial pesticide and herbicide savings while improving its cost and environmental effectiveness.

## Conclusion

In this study, an optimized semi-supervised GAN-based framework was developed for crops and weeds classification. In the developed framework, a fully convolutional multiclass classifier was employed. Instead of the conventional discriminator, the SoftMax function was employed in the final layer of the discriminator to enable classification to predict input data into two classes (crops and weeds) or a fake sample. The developed method was evaluated on the quadcopter’s dataset for two different crop fields, which led to the conclusion that the developed method achieved high accuracy for classification on the imagery of the crops in the early growth stage, where detection of weed was a challenging task due to similar spectral and appearance characteristics. The experimental results also proved that the developed framework could perform better at a higher unlabeled rate than conventional supervised learning techniques. The developed framework achieved an accuracy of almost 90% for both the croplands (pea and strawberry) and a train time of 48.96 sec, utilizing a minimum labeled rate of 20%, where other methods were able to achieve a maximum of (87.13%). The developed method was evaluated on images collected at a fixed 2m altitude, and as future work, the framework should be evaluated on images collected at different heights. Further studies on integrating the system into the Real-Time UAV for crop and weed classification are in progress.
